# A184 DISCOVERY OF POTENTIAL LGR5 LIGANDS

**DOI:** 10.1093/jcag/gwae059.184

**Published:** 2025-02-10

**Authors:** F Gendron, G Arguin, J Acosta Montalvo, P Boudreau

**Affiliations:** Immunology and Cell biology, Universite de Sherbrooke, Sherbrooke, QC, Canada; Immunology and Cell biology, Universite de Sherbrooke, Sherbrooke, QC, Canada; Immunology and Cell biology, Universite de Sherbrooke, Sherbrooke, QC, Canada; Immunology and Cell biology, Universite de Sherbrooke, Sherbrooke, QC, Canada

## Abstract

**Background:**

LGR5, an intestinal stem cell marker, is crucial in mediating R-spondin (RSPO) signaling, supporting canonical WNT signaling for tissue development and renewal. This pathway is pivotal in gastrointestinal (GI) disease, assisting wound healing and playing a role in cancer progression and chemoresistance. While modulating the WNT pathway holds therapeutic potential, directly targeting it risks disrupting cell differentiation and organ function. LGR5, whose activity complements but does not fully overlap with the WNT pathway, represents a safer target for therapeutic intervention.

**Aims:**

This study seeks to identify novel ligands for LGR5, focusing on bioactive molecules in the gut lumen and secreted soluble factors from epithelial cells. In addition, RSPO-derived peptidomimetics were designed and assessed as potential LGR5 ligands.

**Methods:**

Luminal lavages from mice intestines were subjected to size-exclusion chromatography, and their activity was tested using impedance assays in HEK293 cells expressing human LGR5 (HEK293/LGR5). Active fractions were further analyzed using pull-down assays and LC-MS/MS to identify LGR5 interacting proteins. Media from cultured IECs were also processed and evaluated for potential LGR5 ligands using LC-MS/MS. Protein-protein interactions (PPIs) were confirmed via immunoprecipitation (IP). Finally, RSPO peptidomimetics based on the FU2 domain, known to bind LGR5, were tested for their ability to modulate β-catenin activity through luciferase reporter assays.

**Results:**

An isoform of Urotensin II (iUTS2) from the cell culture media and three proteins from intestinal luminal extracts were identified as potential LGR5 ligands. The interaction between iUTS2 and LGR5 was confirmed through IP. As a proof of concept, six RSPO peptidomimetics were designed and tested. Although none of the peptides fully replicated RSPO1 activity, three were found to enhance the effect of RSPO1 on WNT3a-induced β-catenin activity, indicating their potential as modulators of LGR5.

**Conclusions:**

This study presents the first identification of potential endogenous and peptidomimetic LGR5 ligands. Although in the preliminary stages, these findings represent a significant step toward developing selective LGR5 ligands, offering insight into LGR5 receptor functions. Such discoveries hold promise for future LGR5-targeted therapies, which could restore tissue homeostasis in conditions like inflammatory bowel disease or impede cancer progression by preventing cell adaptation and dissemination.

This work was funded by a CCC-GIA research grant (2022-2025) and NSERC (RGPIN-2019-05294).

**Funding Agencies:**

CCCNSERC

